# A new personalized vaccine strategy based on inducing the pyroptosis of tumor cells *in vivo* by transgenic expression of a truncated GSDMD N-terminus

**DOI:** 10.3389/fimmu.2022.991857

**Published:** 2022-09-15

**Authors:** Jinrong He, Peng Zheng, Yongjun Chen, Jialong Qi, Chao Ye, Duo Li, Ying Yang, Ying Yang, Qingwen Liu, Yongmao Hu, Xiao Zheng, Weiran Li, Liangqun Hua, Zhongqian Yang, Haoqian Chen, Weiwei Huang, Wenjia Sun, Xu Yang, Qiong Long, Hongmei Bai, Yanbing Ma

**Affiliations:** ^1^Laboratory of Molecular Immunology, Institute of Medical Biology, Chinese Academy of Medical Sciences and Peking Union Medical College, Kunming, China; ^2^Yunnan Digestive Endoscopy Clinical Medical Center, Department of Gastroenterology, The First People’s Hospital of Yunnan Province, Kunming, China; ^3^Department of Acute Infectious Diseases Control and Prevention, Yunnan Provincial Center for Disease Control and Prevention, Kunming, China; ^4^Institute of Medical Biology, Kunming Medical University, Kunming, China; ^5^School of Life Sciences, Yunnan University, Kunming, China; ^6^School of Ethnic Medicine, Yunnan Minzu University, Kunming, China

**Keywords:** GSDMD, pyroptosis, immunogenic cell death, tumor cell vaccine, antitumor immunity

## Abstract

The variability and heterogeneity of tumor antigens and the tumor-driven development of immunosuppressive mechanisms leading to tumor escape from established immunological surveillance. Here, the tumor cells were genetically modified to achieve an inducible overexpression of the N-terminal domain of gasdermin D (GSDMD-NT) and effectively cause pyroptosis under a strict control. Pyroptotic tumor cells release damage-associated molecular patterns (DAMPs) and inflammatory cytokines to promote the maturation and migration of bone marrow-derived dendritic cells (BMDCs). Furthermore, local tumor delivery, and preventive or therapeutic subcutaneous immunization of the modified cells, followed by the induction of GSDMD-NT expression, significantly stimulated both the systemic and local responses of antitumor immunity, and reprogrammed the tumor microenvironment, leading to the dramatic suppression of tumor growth in mice. This study has explored the application potency of inducing the pyroptosis of tumor cells in the field of tumor immunotherapy, especially for developing a new and promising personalized tumor vaccine.

## Graphical Abstract

Schematic representation of a personalized tumor vaccine strategy based on inducing GSDMD-NT genetically engineered tumor cells to induce pyroptosis and elicit antitumor immune responses. Tumor cells were genetically engineered to inducibly overexpress GSDMD-NT and induce pyroptosis as a novel personalized tumor cell vaccine. After inoculation of mice with the cells, GSDMD-NT was induced to be expressed by providing drinking water with doxy, and it oligomerized on the cell membrane to form pores. Cell swelling and pyroptosis occur with the release of inflammatory molecules, and danger signals including HMGB1, ATP, LDH, CART, and HSP70/90. These DAMPs stimulate the migration and maturation of DCs, and promote the production of tumor-specific CD8+ T cells and IFN-g, accompanied by downregulated systemic myeloidderived suppressor cells (MDSCs) and regulatory T cells (Tregs) responses and remodeling of the tumor microenvironment. Finally, tumor growth was significantly suppressed.

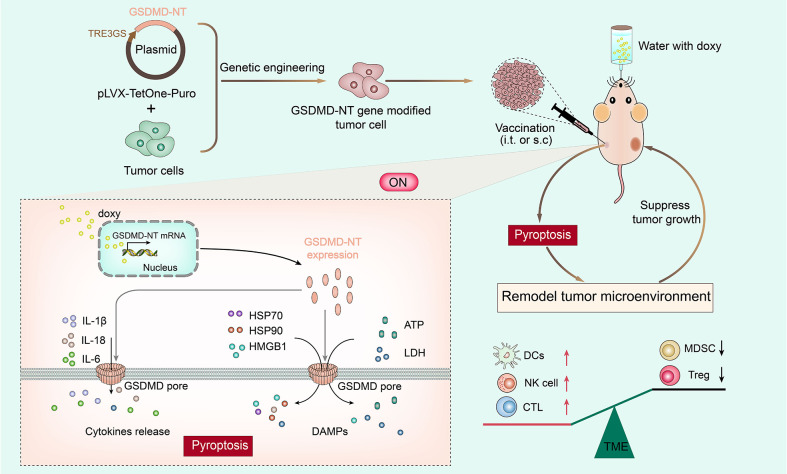

## Highlights

1. GSDMD-NT expression induces tumor cell pyroptosis and ICD mediator release.
2. Pyroptosis-induced tumor cells promote DC migration and maturation.
3. Local tumor delivery of pyroptosis-inducible cells successfully modified the TME.
4. Immunization of the cell vaccine elicits systemic and local antitumor immunity.

## Introduction

In recent years, the incidence and mortality of cancers have increased rapidly, and cancer has become one of the most important causes of death worldwide (1). Traditional surgery, radiotherapy and chemotherapy for tumor treatment usually fail to prevent tumor recurrence (2, 3). Accordingly, achieving continuous elimination of cancer cells through stimulating the immune system and rebuilding effective immunological surveillance is important and promising. The principle of a tumor vaccine is, through providing effective antigens and powerful immune stimulators, to elicit tumor-specific cellular immunity and help the body recover the capability of recognizing and clearing tumor cells. However, immune suppressive and evading mechanisms developed by tumors significantly limit the induction and function of antitumor effector cells.

Pyroptosis is a kind of programmed cell death (PCD) mediated by pore-forming proteins including gasdermin A, B, C, D, and E (4–6). The pyroptosis of tumor cells can release danger signals and inflammatory components, triggering powerful antitumor immunity (7, 8). The gasdermin D (GSDMD) protein was first identified in 2015 as a key effector molecule in the apoptosis process (9). Once cleaved by caspase-1 or caspase-4/5/11 (10), (11), active N-terminal domain of GSDMD (GSDMD-NT) will oligomerizes in the cell membrane to form approximately 18 nm pores, causing cell swelling and cell membrane rupture and releasing inflammatory molecules and cellular contents (9, 12, 13). Pyroptotic cells present many kinds of “find me” and “eat me” danger signals to stimulate immune cells. The release of the nucleoprotein high-mobility group box 1 (HMGB1) protein promotes dendritic cell uptake, processing and presentation of antigens through interaction with Toll-like receptor 4 (TLR4)( 14, 15). Extracellular adenosine-5’-triphosphate (ATP) also serves as an effective “find me” signal (16, 17) and promotes recruitment, subsequent tumor antigen uptake, antigen presentation (18, 19), maturation (20), and homing of DCs (21). ATP binds to P2X or P2Y purine receptors expressed on DCs (22) and thereby activates the NLR family pyrin domain containing three (NALP3)/ASC/inflammasome pathways, leading to the release of immune modulators such as IL-1β. Heat-shock proteins 70/90 (HSP70/90) are usually located in the intracellular compartment and have the function of protecting cells under stress, and extracellular release of HSP70/90 provides signals for immune system activation, and it acts as a carrier for peptide antigen exposure (23). In addition, exposed calreticulin (CALR) on the pyroptotic cell surface, provides an “eat me” signal to promote the phagocytosis of professional antigen presenting cells, including dendritic cells (DCs) and macrophages (24, 25).

Chemotherapeutic drug-induced pyroptosis depends on activation of pyroptosis signaling pathway and expression abundance of effector molecules, and its clinical application for the induction of pyroptosis may be limited due to drug resistance and side effects (26, 27). Previous studies have described that heterogenous expression of GSDMD-NT by an inducible system bypassed the upstream regulatory events that naturally lead to pyroptosis and was sufficient to induce pyroptosis in various cell models (28, 29). In this study, we genetically modified tumor cells to produce inducible overexpression of GSDMD-NT to provide a generally applicable induction method for tumor cell pyroptosis. After inoculation, the controllable induction of pyroptosis can be safely and simply achieved by providing drinking water with a tetracycline analog, doxycycline (doxy). We showed that DAMPs released by pyroptotic cells effectively stimulated the migration and maturation of BMDCs *in vitro*, and promoted systemic and local in tumor anti-tumor effector T cell responses. In grafted tumor models, local delivery into tumors or subcutaneous immunization (both preventive and therapeutic) of the modified cells showed that the induction of tumor cell pyroptosis produced significant effects on suppressing tumor growth. This study proposes a novel strategy for developing an effective personalized tumor vaccine.

## Results

### Inducible expression of genetically modified GSDMD-NT causes pyroptosis in TC-1/4T1/CT26 tumor cells

The well-known executor causing cell pyroptosis is the active domain of the gasdermin protein family, with a pore-forming function (5, 30). At present, six homologous genes of gasdermin family members in humans have been identified, namely, GSDMA, GSDMB, GSDMC, GSDMD, GSDME (DFNA5) and Pejvakin (DFNB59) (31–33). Expression of the N-terminal domains of all gasdermins except for pejvakin is sufficient to directly induce lytic and inflammatory cell death (13, 28). To investigate the immunological characteristics of pyroptotic tumor cells and their potency to be developed as a personalized tumor vaccine, we explored a generally applicable strategy for inducing pyroptosis of tumor cells utilizing the inducible Tet-On systems for the expression of the key downstream effector protein GSDMD-NT (1~276 a.a.) (9, 28, 29, 34), which is independent of any upstream receptor recognition or caspase activation.

**Figure 1:**
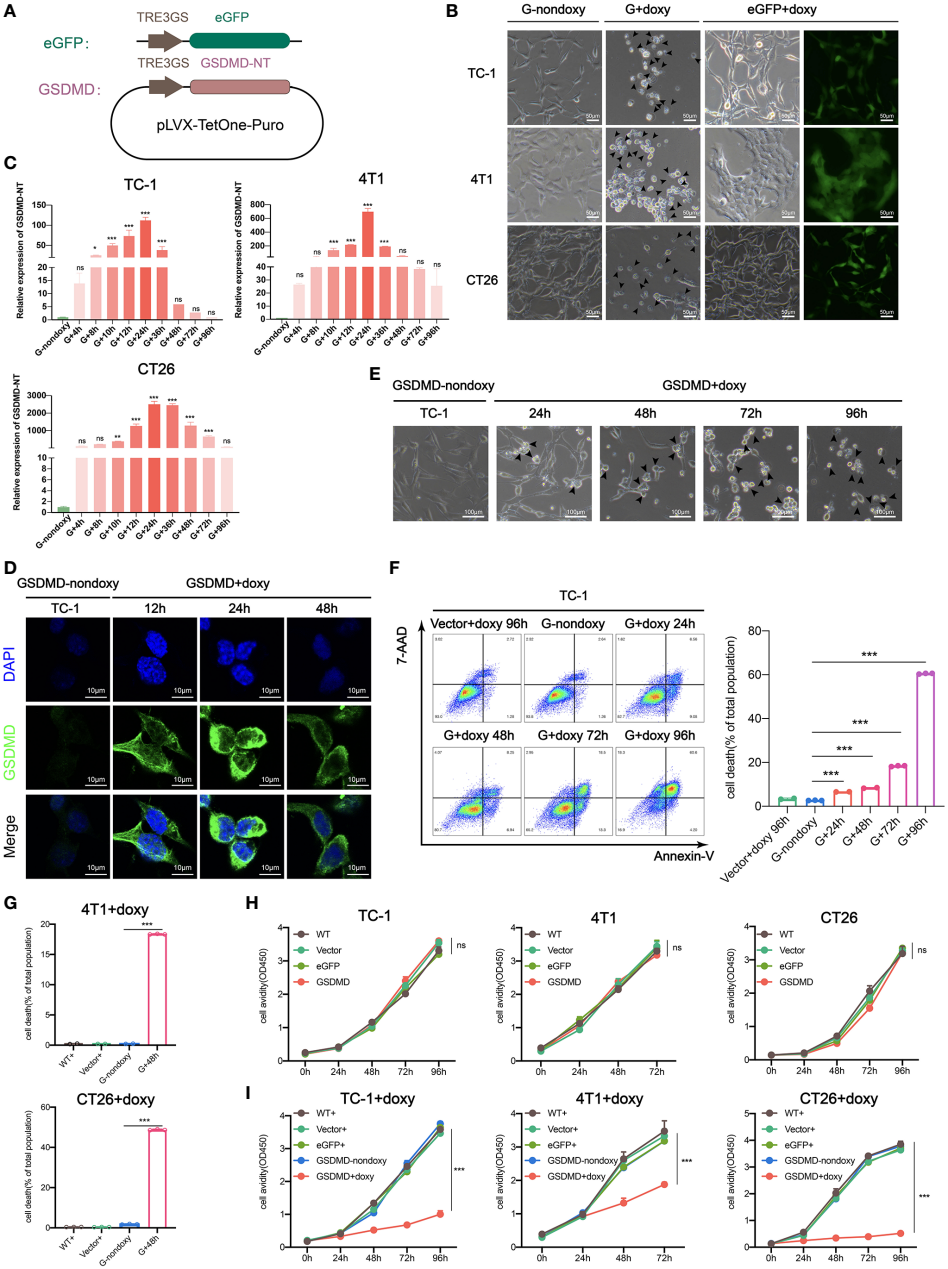
Inducible expression of genetically modified GSDMD-NT causes pyroptosis in TC-1/4T1/CT26 tumor cells. **(A)** Schematic presentation of the Tet-On-inducible GSDMD-NT or eGFP expression constructs. **(B)** Representative images of GSDMD-NT or eGFP expressing cells after 48 hours of incubation with doxy. Arrowheads indicate pyroptotic cells. Scale bar, 50μm. GSDMD or G-nondoxy indicates GSDMD-NT gene-modified cells without doxy induction. **(C)** Histogram analysis of the dynamic expression of GSDMD-NT in TC-1/4T1/CT26 tumor cells measured by RT-qPCR (n=3). G+ indicates induction of GSDMD-NT gene-modified cells by doxy. **(D)** Immunofluorescent staining was performed after incubation with doxy for various durations in TC-1 cells. Blue: DAPI-stained nuclei, green: GSDMD-NT detected with FITC-labeled anti-GSDMD antibodies. Scale bar, 10 μm. **(E)** The classical pyroptosis-like cell morphological changes including swelling in doxy-induced GSDMD-NT-modified TC-1 cells. Scale bar, 100 μm. **(F)** Representative flow cytometry pseudocolor dotplots of 7-AAD- and Annexin V-stained GSDMD-NT expressing TC-1 cells after incubation with doxy (left), vector+doxy (representing represents vector-transfected cells induced with doxy) for 96 h as a homotype control, and G-nondoxy as a blank control. Histogram analysis of dynamic cell death in 7-AAD and Annexin V double-positive cells (right) (n=3). **(G)** Histogram analysis of flow cytometry results of 7-AAD and Annexin V double-positive 4T1 and CT26 cells at 48 hours after incubation with doxy (n=3). **(H)** Proliferation curve of TC-1/4T1/CT26 cells stably transfected with vector, eGFP, or GSDMD-NT and wild-type (WT) by CCK-8 assays (n=5). **(I)** The toxicity of doxy was detected by cell proliferation avidity curve. Doxy was added to vector+, eGFP+, GSDMD-NT+ and wild type (WT+) TC-1/4T1/CT26 cells, and GSDMD-nondoxy was used as a control, “+” represents doxy induction (n=5). **(C, F, G–I)** Graph shows means ± SD; One-way ANOVA; *p value < 0.05, **p value < 0.01, ***p value < 0.001, ns represents no significance.

**Figure 2:**
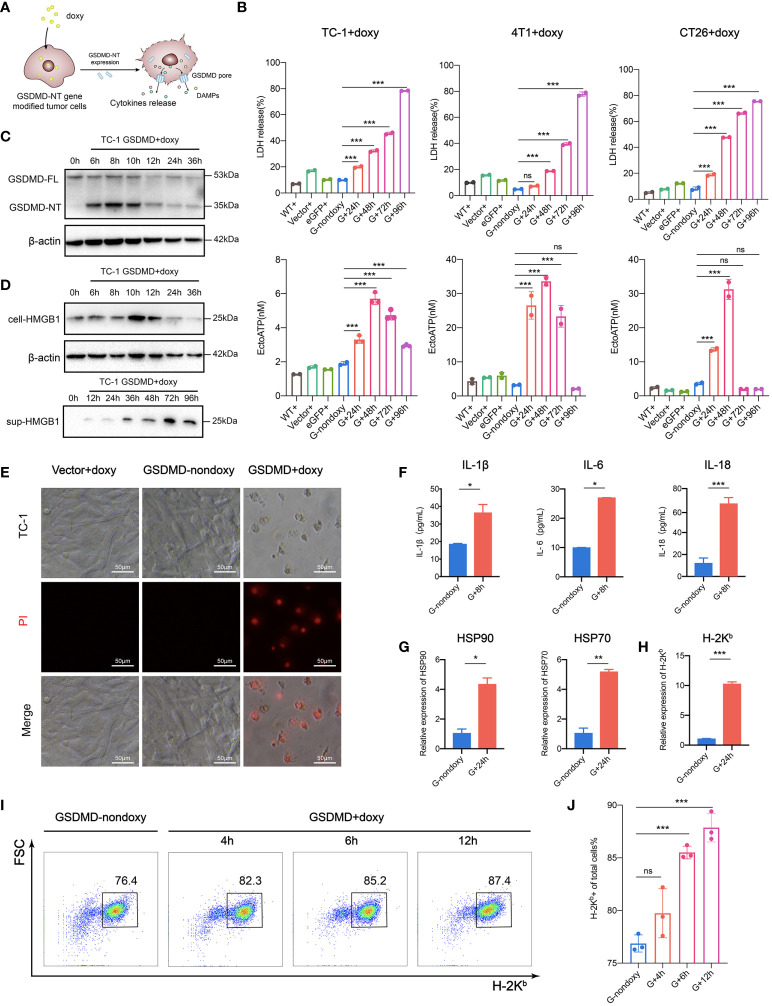
Expression and release of immune mediators in pyroptotic tumor cells. **(A)** Pattern diagram of immunogenic pyroptosis induced by Tet-On-GSDMD-NT gene expression. **(B)** The release of LDH (upper) and ATP (lower) was detected during the process of pyroptosis induction. WT+, Vector+, eGFP+ served as a homotype control to eliminate the nonpyroptotic effects of doxy on cells, and G-nondoxy served as a blank control. **(C, D)** Western blotting showing the expression of GSDMD-NT and **(D)** the expression and release of HMGB1 was induced in genetically modified TC-1 cells by doxy. **(E)** PI uptake assays. The modified TC-1 cells were incubated with doxy for 48 h and observed under a fluorescence microscope 6 h after PI dye was added. Scale bar, 50 μm. **(F)** The release of inflammatory cytokines in the supernatant of pyroptotic tumor cells was detected by ELISA (n=3). **(G, H)** The expression of HSP70, HSP90 and **(H)** H-2K^b^ in the modified TC-1 cells was analyzed by RT-qPCR (n=3). **(I, J)** Representative flow cytometry pseudocolor dotplots and **(J)** histogram analysis of H-2K^b^ expressing cells (n=3) in the dynamic pyroptosis process of the modified TC-1 cells. Graph shows means ± SD; **(B, J)** One-way ANOVA; **(F, G, H)** unpaired Student’s t test, *p value < 0.05, **p value < 0.01, ***p value < 0.001, ns represents no significance.

First, the GSDMD-NT gene was cloned into the lenti-vector plasmid pLVX-TetOne-Puro under the control of the TRE3GS promoter, and the eGFP gene was cloned in parallel as a marker gene (**Figure 1A**). The plasmids lenti-vector, lenti-eGFP and lenti-GSDMD-NT were transduced into mouse tumor cells TC-1, 4T1, and CT26 respectively, followed by puromycin selection. As expected, when detected at 48 hours after the addition of doxy to the medium the expression of eGFP was induced showing clear fluorescence. Transduction of lenti-GSDMD-NT resulted in swelling and round tumor cells, presenting typical pyroptotic cell morphology as compared to the lenti-GSDMD-NT without doxy control (**Figure 1B**). Meanwhile, the dynamic transcription levels of the GSDMD-NT mRNA were measured, which increased with the extension of induction time and reached a maximum at approximately 24 hours after doxy induction (**Figure 1C**). The expression of GSDMD-NT in TC-1 cells was further confirmed by immunofluorescence assays detecting the target protein in the cultured cells, which also validated the membrane location of expressed GSDMD-NT (**Figure 1D**). The dynamic morphological changes in the TC-1 cells were monitored, showing that almost all of the cells swelled up and became round at 72 hours (**Figure 1E**). Accordingly, the TC-1 cells collected at 24 h, 48 h, 72 h and 96 h were subjected to 7-AAD and Annexin V staining for flow cytometry analysis, and the results showed that the percentage of dead cells increased with doxy incubation (**Figure 1F**). Death also occurred in 4T1 and CT26 cells after doxy induction (**Figure 2G**). In brief, with the accumulation of GSDMD-NT expression, the degree of pyroptosis-like morphology changes became stronger, and lytic cell death eventually occurred. Furthermore, it was shown that there was no difference in the proliferation avidity between the genetically modified tumor cells without doxy induction and the wild-type cells (**Figure 1H**). We also tested the cytotoxicity of doxy itself on cells, and the results showed that the dose used in the experiment did not affect the cell proliferation. It was clear that the toxicity presented in doxy-induced GSDMD-NT gene-modified tumor cells was completely dependent on the expression of GSDMD-NT (**Figure 1I**). In general, we successfully created a system for inducing pyroptosis in TC-1, 4T1, and CT26 tumor cell lines, in which the expression of GSDMD-NT was strictly controlled by the inducer doxy.

### Expression and release of immunogenic mediators in pyroptotic tumor cells

Pyroptosis is a newly recognized form of ICD that imparts high immunogenicity to tumor cells. It improves the recognition of tumor cells by the immune system by releasing DAMPs that are normally hidden within living cells and it specifically stimulates antitumor immune responses to eliminate cancer cells (35–40). The “find me” signal molecules released by immunogenically dead cells bind to their own receptors, recruiting and activating immunecells; in addition, the “eat me” signals promote tumor cells to be taken up by professional phagocytes, leading to enhanced antigen processing and presentation to T lymphocytes. The ICD occurring in some of tumor cells has the capability of guiding the immune system to track, recognize and kill other more cancer cells (25, 37, 41). In this study, we employed a new strategy by cutting off the upstream signaling and directly expressing the downstream effector molecule GSDMD-NT to induce pyroptosis. To clarify the immunological characteristics of pyroptosis induced in this way, we carefully examined the expression and release of possible immunogenic mediators (42) (**Figure 2A**). The tumor cells were incubated with doxy for different time durations, and they released an increasing amount of lactate dehydrogenase (LDH) over time (**Figure 2B** upper). ATP release was induced to the highest level at 48 hours in TC-1, CT26, and 4T1 cells (**Figure 2B** lower). Western blotting showed that GSDMD-NT was quickly induced by doxy (**Figure 2C** and **Figure S1**). The expression and progressive secretion and accumulation of the HMGB1 in the supernatant were found in doxy-induced TC-1 cells (**Figure 2D**). Meanwhile, PI uptake assays in TC-1 cells showed that the degree of damage to the cell membrane was severe at 48 hours after doxy induction (**Figure 2E**). The release of the inflammatory cytokines was found to be significantly increased in GSDMD-NT-induced pyroptotic cells (**Figure 2F**). In addition, real time qPCR showed that the transcription levels of HSP70 and HSP90 were upregulated (**Figure 2G**). The expression of MHC class I molecules is important for presenting antigens and eliciting the tumor-specific immune responses (43), and in qPCR analysis H-2K^b^ was higher in pyroptotic tumor cells (**Figure 2H**). Furthermore, the increased expression of H-2K^b^ was supported by dynamically analyzed H-2K^b^-positive cells with flow cytometry (**Figures 2I, J**). It was reported that the ligand-dependent or drug-inducing pyroptosis can trigger the secretion of the inflammatory cytokines IL-1β and IL-18 to stimulate the immune system (44, 45). In a summary, GSDMD-NT gene-modified and doxy-induced tumor cells have similar immunological characteristics to those of classical approach-induced pyroptotic cells, indicating that our strategy provides an efficient and universal method for ICD induction and thus has the potential to be used for the development of tumor cell vaccines.

### Immunogenic pyroptotic tumor cells promote the migration and maturation of BMDCs

**Figure 3:**
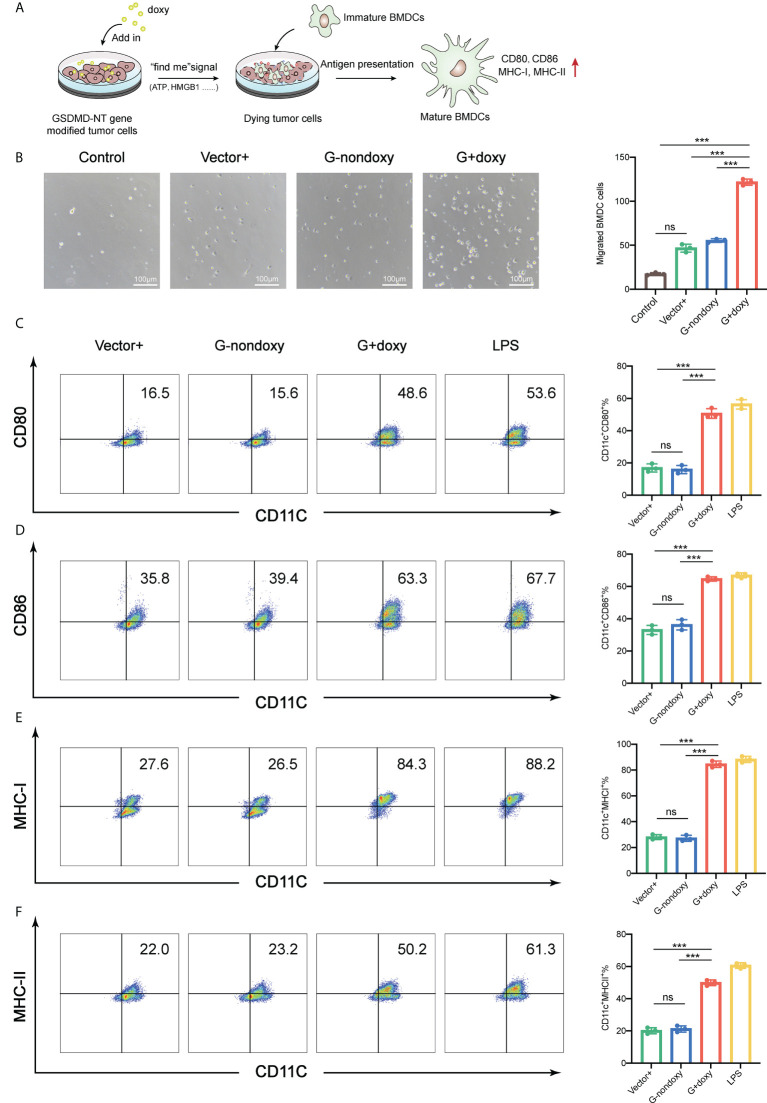
Immunogenic pyroptotic tumor cells promote the migration and maturation of BMDCs. **(A)** Pattern of immunogenic pyroptotic tumor cells promoting BMDC migration and maturation. **(B)** Transwell migration assays showed that the supernatant derived from the modified TC-1 cells incubated with doxy for 24 h induced the migration of BMDCs. Representative images and statistical analysis are presented. The “control” served as background contrast. Scale bar, 100 μm. **(C–F)** Representative flow cytometry pseudocolor dotplots for mature marker CD80-, CD86-, MHC-I-, and MHC-II-expressing cells in CD11c^+^ BMDCs and the corresponding statistical analysis. The BMDCs were incubated with pyroptotic TC-1 cells for 18 h. LPS served as a positive control. **(B–F)** Graph shows means ± SD, One-way ANOVA, ***p value < 0.001, ns represents no significance.

As mentioned above, doxy-treated GSDMD-NT cells can release the danger signal molecules (ATP and HMGB1) to recruit and activate DCs. To further support this hypothesis, a Transwell migration assay was performed with the supernatant of pyroptotic tumor cells added to the lower chamber and the BMDCs were placed into the upper chamber, or BMDCs was directly incubated with pyroptotic tumor cells (**Figure 3A**). After incubation for 6 hours, BMDCs were recruited by the supernatant of pyroptotic cells but not the normal cell supernatant, which served as a control (**Figure 3B**). At the same time, the GSDMD-NT-TC-1 cells and BMDCs were cocultured at a ratio of 1:10 for 24 hours after adding the doxy. They were analyzed by flow cytometry for the expression of costimulatory molecules CD80/CD86 and antigen-presenting molecule MHC I and MHC II. Their expression on the surface of the BMDCs was upregulated as compared to the controls, including vector-transfected cells with doxy induction and GSDMD-NT cells without doxy induction. These results indicated that pyroptotic tumor cells effectively stimulated BMDC migration and promoted their maturation activation (**Figures 3C–F**).

### GSDMD-NT overexpression induces the complete clearance of the inoculated genetically modified tumor cells in mice

**Figure 4:**
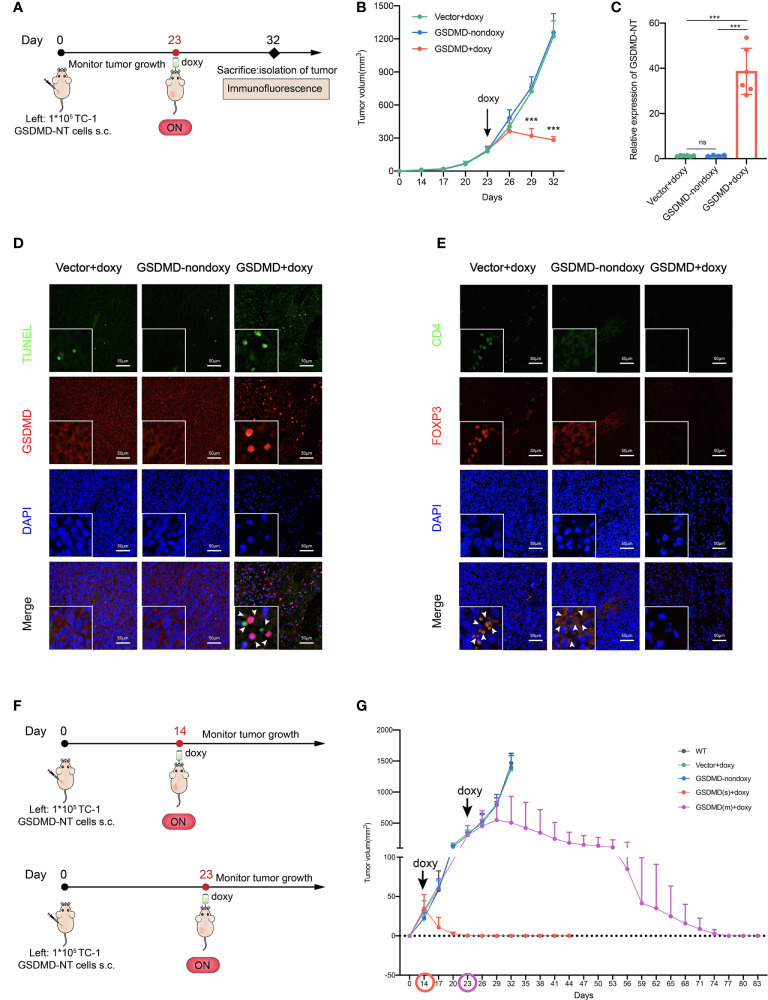
GSDMD-NT overexpression induces the complete clearance of the inoculated genetically modified tumor cells in mice. **(A)** The protocol for evaluating the growth of genetically inoculated tumors itself after inducing GSDMD-NT expression in a TC-1 tumor model. **(B)** The monitoring of tumor growth (n=3). **(C)** The expression of GSDMD-NT in the tumor tissues measured by RT-qPCR (n =6). On Day 32, the mice were sacrificed and the tumors were isolated. **(D)** TUNEL analysis (green) and immunofluorescence analysis of GSDMD-positive cells (red). DAPI was used for nuclear staining (blue). Scale bar, 50 μm. **(E)** Immunofluorescence analysis of tumor-infiltrating Tregs, using anti-CD4 and anti-Foxp3 antibody double staining represents Tregs, and DAPI was used for nuclear staining (blue). Scale bar, 50 μm. **(F)** The protocol for evaluating the potency of inducing GSDMD-NT-mediated pyroptosis, initializing the induction of GSDMD-NT expression at different tumor sizes. **(G)** The monitoring of tumor growth. GSDMD(s) and GSDMD(m) represent drinking doxy when the tumor volume reached to 20-50 mm^3^ on Day 14 or 200-500mm^3^ on Day 23, respectively (n=5). Graph shows means ± SD, **(B)** Two-way ANOVA; **(C)** One-way ANOVA; ***p value < 0.001, ns represents no significance.

The immunological properties of GSDMD-NT-mediated pyroptotic tumor cells were analyzed *in vitro* as described above. Next, the genetically modified tumor cells were inoculated into mice to test whether artificial control of GSDMD-NT expression and pyroptosis occurrence induced by supplementing their drinking water with doxy could abolish the development of tumors from the inoculated cells (**Figure 4A**). Notably, the results showed that the growth of inoculated tumor cells was significantly suppressed in the GSDMD-NT-modified TC-1 tumor-bearing mice with the induction of doxy in drinking water, as compared to that in the mice drinking water without doxy or that in the vector-transfected tumor-bearing mice (**Figure 4B**). These results indicated that the Tet-On system was controllable *in vivo*, and doxy itself didn’t affect tumor growth compared with the two control groups. Furthermore, the expression of GSDMD-NT in tumor tissue was confirmed by RT-qPCR after the doxy induction (**Figure 4C**). Then, a TUNEL staining was performed to analyze the pyroptotic cells in tumor tissues, and a specific labeled antibody was used to mark the expression of GSDMD molecules, and both of them were induced by doxy treatment (**Figure 4D**). Immunofluorescence staining showed that doxy treatment decreased the infiltration of immunosuppressive cells Tregs in tumor tissues (**Figure 4E**). The above results suggested that doxy-induced GSDMD-NT overexpression in TC-1 tumor bearing mice led to pyroptosis of tumor cells and remodeled the tumor immunosuppressive microenvironment. To further evaluate the antitumor potencies of inducing pyroptosis in tumor cells, GSDMD-NT expression was induced at different tumor sizes, *i.e.*, 20-50 mm^3^ and 200-500 mm^3^ (**Figure 4F**). The induction of pyroptosis at 20-50 mm^3^ completely eradicated the tumors after approximately one week of drinking doxy water, and the induction initialed at 200-500 mm^3^ had the capability of reversing the tumor growth to tumor-free status approximately 50 days later (**Figure 4G**). In addition, we also conducted a similar experiment in an orthotopic 4T1 breast tumor model, and the induction of pyroptosis significantly promoted antitumor immune responses and suppressed tumor growth (**Figure S2**). Together, the results indicated that the *in vivo* induction of GSDMD-NT-mediated pyroptosis was effective enough to completely eliminate the established tumors from the inoculated genetically modified tumor cells.

### Preventive immunization with the novel pyroptosis induction-based tumor cell vaccine significantly inhibits the growth of grafted tumors in mice

**Figure 5:**
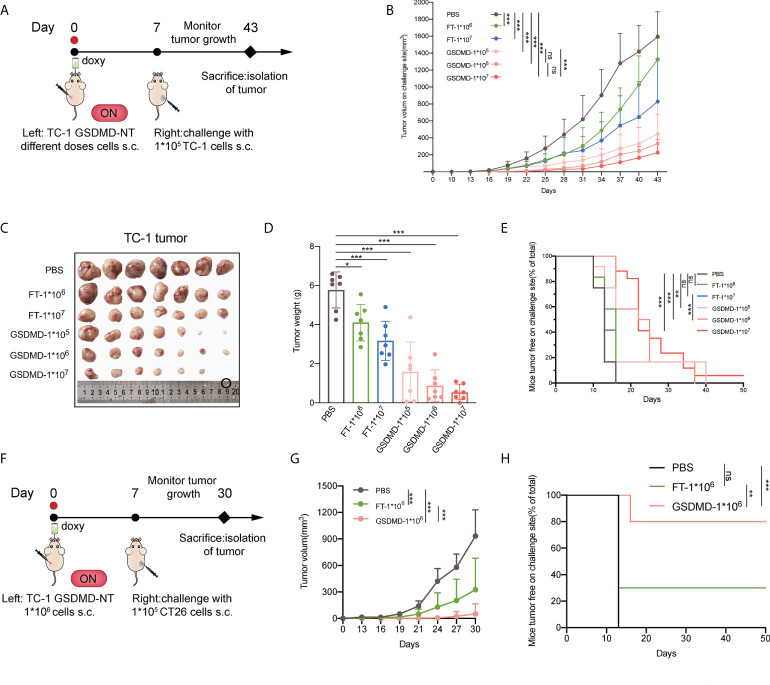
Preventive immunization with the novel pyroptosis induction-based tumor cell vaccine significantly inhibits the growth of grafted tumors in mice. **(A)** The protocol of the preventive immunization strategy for evaluating the potency of the immunity elicited by the pyroptotic TC-1 cells against the challenge of homogenous tumors in C57 mice (n=12). **(B)** Tumor growth curves by group (n=12). The repeated freeze-thaw lysis of tumor cells (FT cells) and PBS were used to replace the pyroptotic cells as blank controls. **(C)** The size of the isolated tumor mass (n=7). **(D)** The weight of the isolated tumor mass (n=7). **(E)** The percentage of the tumor-free mice (n=12). **(F)** The protocol of the preventive immunization strategy for evaluating the possible effects of pyroptotic TC-1 cells against the heterogeneous challenge of CT26 tumors in BALB/c mice (n=10). **(G)** Tumor growth curves by groups. **(H)** Percentage of the tumor-free mice. **(B, D, G)** Graph shows means ± SD, One-way ANOVA; **(E, H)** Log-rank test, *p value < 0.05, **p value < 0.01, ***p value < 0.001, ns represents no significance.

To assess the potency of employing pyroptotic tumor cells as a novel vaccine, the modified tumor cells were injected as a tumor-preventive strategy simultaneously with the induction of GSDMD-NT expression by putting doxy in the drinking water. The mice were then subjected to contralateral subcutaneous challenge with wild-type tumor cells one week later (**Figure 5A**). The results showed that regardless what the vaccination dose of the modified tumor cells is sufficient to prevent the growth of the inoculated wild-type tumor (**Figures 5B** and **S3**). Although the repeated freeze-thaw cell lysate (FT) also inhibited the growth of tumors as compared to the unvaccinated control (the mice only received PBS), the GSDMD-NT-expressing tumor cells presented a better effect, indicating that the pyroptotic cells produced stronger antitumor immunity. These results were supported by the data about the sizes of the isolated tumor masses (**Figure 5C**), the weight of the tumor masses (**Figure 5D**), and the tumor free rate of the mice (**Figure 5E**). In brief, GSDMD-NT expression induced pyroptotic TC-1 cells to significantly or even completely suppress the tumor growth in mice challenged with wild-type TC-1 tumor cells. To clarify the possible contributions of the innate immunity elicited by ICD mediators and molecules related to pyroptotic cells, their possible cross-immune protection effects on heterogeneous CT26 tumors were investigated. Similar to the previous experiments,1×10^6^ GSDMD-NT-TC-1 tumor cells were subcutaneously inoculated on the left side of BALB/c mice. After drinking water with doxy for one week, the mice were challenged with CT26 tumor cells on the right side at a dose of 1×10^5^ cells/mouse (**Figure 5F**). The data showed that the pyroptotic TC-1 tumor cell vaccine (GSDMD-NT-TC-1) significantly inhibited the growth of CT26 tumors (**Figures 5G** and **S4**) and protected 80% of the mice from forming a tumor (**Figure 5H**), indicating that the pyroptotic cell vaccine can elicit strong innate immunity and not just tumor antigen-specific systemic antitumor effects.

### Local inoculation with the pyroptosis-inducible tumor cells suppresses the growth of established TC-1 tumors and significantly improves the antitumor cellular immune responses

**Figure 6:**
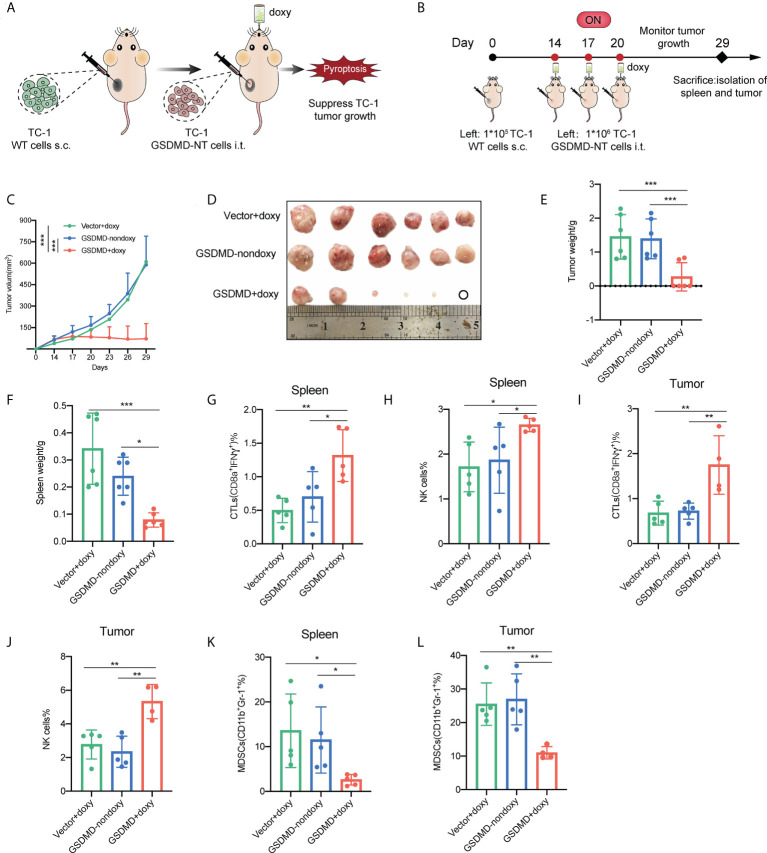
Local inoculation with the pyroptosis-inducible tumor cells suppresses the growth of established TC-1 tumors and significantly improves the antitumor cellular immune responses. **(A)** Schematic representation of the treatment of TC-1 xenograft tumors by local inoculation of the pyroptosis-inducible tumor cells. **(B)** The experimental protocol (n=6). **(C)** Tumor growth curves by groups. **(D)** The size of the tumor mass. **(E)** The weight of the tumor mass. **(F)** The weight of the spleen. **(G–L)** CTLs (IFNγ^+^CD8α^+^), natural killer cells (NK1.1^+^), and MDSCs (CD11b^+^Gr-1^+^) in the spleen **(G, H, K)** and tumor tissues **(I, J, L)** were analyzed by flow cytometry (n=5). **(C, E– L)** Graph shows means ± SD, One-way ANOVA, *p value < 0.05, **p value < 0.01, ***p value < 0.001.

Pyroptotic tumor cells have already shown the significant antitumor potency through a preventive immunization. However, the strategy escaped from the immune effects by employing immunosuppressive mechanisms developed by tumor and doesn’t simulate very well the clinical settings of a vaccine application. Here, the pyroptotic tumor cells were inoculated locally into established TC-1 tumors (**Figures 6A, B**). The results showed that the local inoculation of GSDMD-NT gene modified tumor cells with the induction of pyroptosis by drinking with doxy water significantly suppressed the growth of fully established tumors and in some cases, led to complete eradication of the tumor (**Figures 6C** and **S5**). Compared to the controls that received vector cells with induction or GSDMD-NT cells without induction, the induction of pyroptosis in GSDMD-NT cells significantly reduced the size (**Figure 6D**) and the weight (**Figure 6E**) of the isolated tumor masses, and the weight of the spleen (**Figure 6F**). Lymphocytes were isolated from the spleen and tumor tissues for flow cytometry analysis. The data showed that the responses of anti-tumor immune effector cells cytotoxic T lymphocytes (CTLs) (**Figures 6G, I**) and NK cells (**Figures 6H, J**) significantly increased in both spleen and tumor tissues, while the response of immunosuppressive cells MDSCs decreased (**Figures 6K, L**). These results indicated that local induction or delivery of pyroptotic tumor cells into tumors was able to regulate the tumor microenvironment (TME) and elicit anti-tumor innate and adaptive immune responses, and thereby inhibiting the growth of established tumors.

### Therapeutic immunization with the novel pyroptotic tumor cell vaccine suppresses the growth of established TC-1 tumors and significantly improves the antitumor cellular immune responses

**Figure 7:**
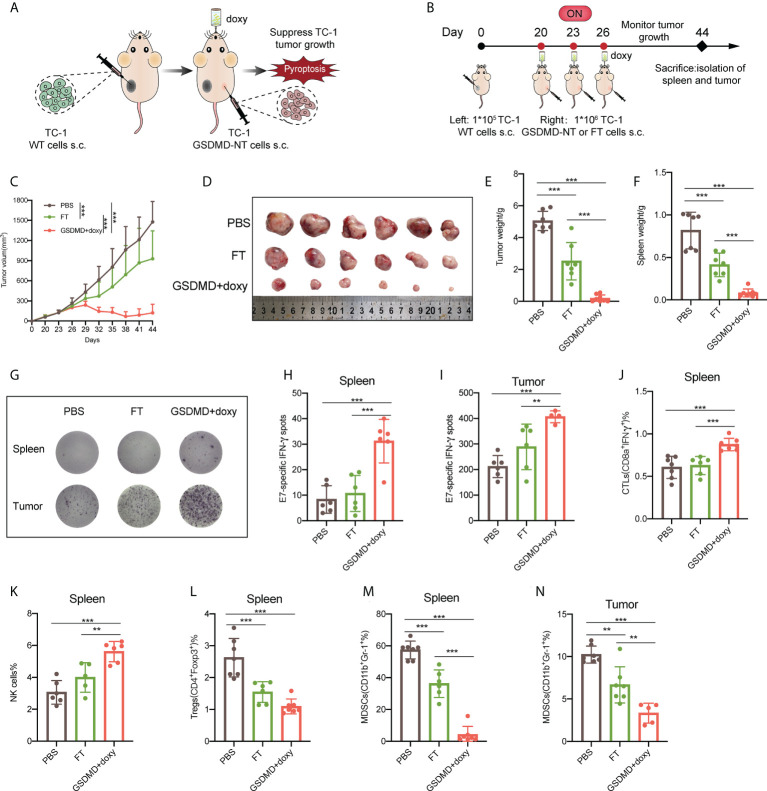
Therapeutic immunization with the novel pyroptotic tumor cell vaccine suppresses the growth of established TC-1 tumors and significantly improves the anti-tumor cellular immune responses. **(A)** Schematic representation of the treatment of TC-1 xenograft tumors by therapeutic immunization with the pyroptosis-inducible tumor cells. **(B)** The experimental protocol (n=8). **(C)** Tumor growth curves by groups. **(D)** The size of the tumor mass (n=6). **(E)** The weight of the tumor mass. **(F)** The weight of the spleen. **(G)** Representative images of E7-specific IFN-γ-expressing lymphocytes isolated from the spleen and tumor tissues (n=6). **(H, I)** Statistical analysis of the ELISPOT results of E7-specific IFN-γ-expressing lymphocytes in the spleens and tumors. **(J–M)** Flow cytometry analyses of lymphocytes isolated from the spleen, including CTLs (IFNγ^+^CD8α^+^) **(J)**, natural killer cells (NK1.1^+^) **(K)**, Tregs (CD4^+^Foxp3^+^) **(L)**, and MDSCs (CD11b^+^Gr-1^+^) **(M)**. **(N)** MDSCs (CD11b^+^Gr-1^+^) detected by flow cytometry in tumors. **(C, E, F, H–N)** Graph shows means ± SD, One-way ANOVA, **p value < 0.01, ***p value < 0.001.

To further explore the application of the novel pyroptotic tumor cell vaccine in a clinical setting, in this study, a therapeutic vaccination strategy was employed. As described above, a TC-1 tumor model was fully established with the tumor volume reaching to 40~100 mm^3^, and then the novel pyroptotic tumor cell vaccine was injected subcutaneously into the contralateral site on the back (**Figures 7A, B**). Tumor growth was monitored, and in comparison, with the control of tumor cell lysis vaccine (FT), the pyroptotic tumor cell vaccine more significantly suppressed the growth of established tumors (**Figures 7C**, **S6**). This result was consistent with the size (**Figure 7D**) and weight (**Figure 7E**) of the isolated tumor masses. In addition, the weight of the spleen was decreased in the mice receiving the pyroptotic cell vaccine (**Figure 7F**), implying the suppressed proliferation of lymphocytes due to the reduced tumor burden caused by vaccination. Furthermore, the characteristics of the systemic and local immune responses elicited by the vaccination in tumor-bearing mice were investigated. ELISPOT assays showed that IFN-γ secretion of lymphocytes in both spleen and tumor tissues was significantly increased in the pyroptotic cell group in comparison with those in the controls that either received PBS or freeze–thaw cell lysates in place of the vaccine, after stimulation with the specific E7 peptide *in vitro* (**Figures 7G–I**). The results of the enhanced antitumor immunity were further supported by flow cytometry analysis, which showed that the responses of CTLs (**Figure 7J**) and NK cells in the spleen (**Figure 7K**) were increased, while the responses of immunosuppressive Tregs (**Figure 7L**) in spleen and MDSCs (**Figures 7M, N**) cells were decreased significantly in the spleen and tumor. In general, the novel pyroptotic tumor cell vaccine presented a strong capacity to stimulate antitumor immune responses, downregulate immunosuppressive systemic responses and the tumor microenvironment, and produce a robust inhibitor effect on the growth of the established tumor.

## Discussion

Tumor vaccines are a promising immunotherapeutic strategy; however, they generally lack a significant or satisfactory clinical efficacy at present. One of the possible reasons is the tumor-driven development of systemic and local tumor immunosuppressive mechanisms, which limit the generation and functional exertion of vaccine-induced antitumor effector cells (46); another reason may be attributed to the variability and heterogeneity of tumor antigens due to the genomic instability of tumor cells, which produces frequent changes in tumor antigenicity leading to tumor escape from established immunological surveillance (47). Thus, a successful vaccine strategy needs to effectively overcome immunosuppression and elicit the generation of effector cells targeting a broad spectrum of tumor antigens (48), especially with the involvement of neoantigens and personalized antigens. At present, the main tumor vaccine forms include peptide and protein-based vaccines, DNA vaccines, RNA vaccines, viruses or bacterium-vectored vaccines, DC vaccines, or tumor cell vaccines. Among these vaccines, tumor cell-based vaccines are a relatively old and traditional strategy and are thought to produce a certain of treatment efficacy against prostate cancer in the clinic (49). One of its limitations is its weaker capability of eliciting tumor-specific immune responses. However, with the development of new molecular biology technologies and immune responses theories, the vaccine strategies based on engineered tumor cell lines has been becoming more and more promising and clinically practicable. For example, granulocyte-macrophage colony-stimulating factor (GM-CSF)-secreting allogeneic pancreatic tumor cell vaccine (GVAX) has received multiple attractive clinical trials alone or in combination with other treatments (50). Theoretically, tumor cell lines are convenient to be engineered to overexpress specific and personalized tumor antigens/neoantigens and thus empowered to highlight antigen of interest -specific antitumor responses while the tumor cell itself provides multiple tumor antigens and immune stimulators, which further explores the application potentials of a vaccine based on tumor cell lines. In this study, we provide an engineered tumor cell vaccine strategy through inducing pyroptosis of tumor cells and taking advantage of its immunological characteristics to elicit anti-tumor immunity. Besides using cell lines, the tumor cells can also be obtained through isolating from the excised tumor tissues in patients receiving an operative treatment.

Chemotherapy is a major clinical tool for fighting against cancers. In addition to directly killing tumor cells, some chemotherapy drugs, such as anthracycline and platinum drugs, may also stimulate antitumor immunity by causing immunogenic cell death (ICD) of tumor cells, which brings about a dual antitumor effect. ICD-induced tumor cells release damage-associated molecular patterns (DAMPs) as danger signals, including calreticulin (CALR), high mobility group protein 1 (HMGB1), heat shock proteins (HSPs), and adenosine-5’-triphosphate (ATP), to activate the immune system and enhance antitumor immune responses (51). The immunological features indicate that immunization with ICD-induced tumor cells might be a new vaccine strategy that may be able to simultaneously provide multiple or personalized tumor antigens and strong immunostimulatory signals. This strategy has been reported previously in a few studies (52, 53). However, in order for ICD cells to act as a vaccine they need to control the proper “dying” status, allowing the cells to release immune mediators for a necessary time duration. This means that rational control of ICD status for optimized stimulation of the immune system is difficult when using drugs to induce ICD. In addition, the sensitivity and conditions for ICD induction may vary with the sources and types of tumor cells. This is key challenge in developing ICD cells for tumor immunotherapy as a new vaccine strategy.

Pyroptosis is a newly identified ICD mode that is characterized by having a central effector pathway of membrane pore formation mediated by a processed active protein from the GSDM family. It has been demonstrated that the overexpression of full-length GSDME in transplanted tumor cells could function as a vaccine through promoting the cleavage of GSDME into a pore-forming fragment, which causes tumor cell pyroptosis and enhances tumor-associated macrophage phagocytosis and the responses of tumor-infiltrating NK and CD8+ T lymphocytes (54). Another study developed a bioorthogonal system by engineering tumor cells to express a chemically cleavable construct of GSDMA3 to drive pyroptosis-induced inflammation and trigger robust T cell-depend anti-tumor immunity, revealing that less than 15% of tumor cell pyroptosis was sufficient to clear the entire 4T1 mammary tumor graft (55). Both of the studies strongly indicated that it was an attractive idea to take advantage of the immunological features of pyroptosis for developing new tumor immunotherapeutic strategies especially for the design of a tumor cell vaccine. In this study, we developed a new and commonly applicable approach to induce pyroptosis of tumor cells through transgenic modification of the GSDMD-NT gene. The overexpression of GSDMD-NT was strictly controlled in an inducible pattern by the TRE3GS promoter and tetracycline induction system. In addition, only the intracellular GSDMD- NT can cause pyroptosis, and the addition of activated gasdermin outside the cell will not cause membrane lysis (6, 45). Therefore, in this study, the remaining GSDMD-NT after pyroptosis and lysis of GSDMD-NT-expressing tumor cells did not cause damage to nearby tissues and cells. Altogether, our design explored vaccine application of pyroptotic cells, which allows the cells to express enough ICD mediators *in vivo* in a safe way and provides a new cell vaccine platform for further genetic modification with more functional genes.

At the beginning of the study, we demonstrated that overexpression of GSDMD-NT had the capacity to induce pyroptosis in multiple tumor cell lines, including TC-1, 4T1, and CT26. Our unpublished data using chemotherapeutic drugs to induce ICD showed that the three tumor cell lines presented different sensitivity and response features to specific drugs, while in this study, pyroptosis was indued successfully in all the three cells, supporting that GSDMD-NT is a key effector for pyroptosis induction and our strategy is a broadly applicable approach. We further analyzed the expression and release pattern of ICD mediators from GSDMD-NT-mediated pyroptosis, and the results confirmed that it produced similar immunological responses as classical receptor-mediated pyroptosis, although it lacks the direct triggering of upstream signaling in the classical pathway. It’s interesting that the three cell lines showed different dynamics of the pryoptotic induction, evidenced by Annexin V/7-ADD staining and LDH release, which may correlate with very different magnitudes/kinetics of GSDMD-NT mRNA accumulation in Fig 2C. However, it seems that the levels of GSDMD-NT protein didn’t correlate with pryoptotic cell death (Fig 3C and S1), which implied that the modulatory mechanism of membrane pore formation and pyroptosis was not simply associated with GSDMD-NT expression and more pathways might be involved. It was reported that the expression of endogenous GSDME gene in CT26 cells is much higher than that in 4T1 cells (54), and GSDMD-NT proteins can target the mitochondria to promote cytochrome c release and Gasdermin E cleavage by caspase-3 activation to liberate the GSDME-N domain and thus augment the pyroptosis pathway (56). In addition, the post- translational mTOR signaling pathway and reactive oxygen species (ROS) metabolic signaling-involved regulation mechanisms of membrane pore formation probably determine the dynamics of active GSDMs proteins -mediated pyroptosis (29).

We revealed through *in vitro* experiments that GSDMD-NT genetically modified cells can be effectively induced undergo pyroptosis with fully characterized presentation of immunological mediators of immunogenic death. In addition, we measured the kinetics features of cell death after induction with doxy and ensured that the cells undergoing 24 h doxy induction were destined to die even if doxy was withdrawn, which supports to some extent the safety and effectiveness of the strategy. The immunological effects of pyroptotic cells on DCs, including their influences on migration, antigen uptake and processing, and DC maturation, were further explored. Taken together, we concluded that genetically modified tumor cells with inducible GSDMD-NT overexpression demonstrated full ICD characteristics, with potential to be explored as a new tumor cell vaccine.

The first step to evaluate the application prospects of pyroptosis-inducible cell vaccines is to determine whether doxy induction can effectively prevent the development of tumors caused by an inoculated modified tumor cell itself. For this purpose, mice were inoculated with modified cells and induced with drinking doxy water when the tumor consisting of the modified cells was fully established. We found that regardless of whether the tumor sizes had reached 20-50 mm^3^ or 200-500 mm^3^ when the induction of doxy started, the tumors were eliminated completely, although larger tumors did need a longer time for clearance. The results clearly indicated that the inoculated modified tumor cells would not introduce a new tumor *in vivo*, which laid a basis for its application as a vaccine from the point of view of safety and effectiveness. Furthermore, to evaluate the possible efficacy of employing pyroptosis-inducible cells as a vaccine, a preventive immunization procedure was performed in this study. Although this procedure did not mimic a clinical setting very well, it allows us to determine the inherent ability of the novel pyroptotic cell vaccine to elicit antitumor immunity without the influences of tumor-developed immunosuppression mechanisms. Immunization with a pyroptotic cell vaccine significantly suppressed the development of subsequent challenge with homogenous tumors. Our further experiment demonstrated that immunization with a pyroptotic cell vaccine even provided noticeable protection against a challenge with heterogeneous tumors, implying the effects of innate immunity. It is not surprising to find innate cells’ antitumor effects here, since pyroptotic cells release a large amount of danger signals and inflammatory cytokines, which have important contributions to developing innate immunity (57). The above *in vivo* studies laid a foundation for using pyroptotic cells as a new tumor cell vaccine on the basis of the *in vitro* characterization of ICD cells.

The TME plays a key role in tumorigenesis, immune escape and metastasis (58), and the suppressive TME hinders the ability of T cells to eradicate tumor cells (59). Anti-CTLA-4, anti-PD-1/PD-L1 antibody and other immune checkpoint inhibitors (ICIs) have greatly improved the results of tumor treatment in clinical practice (60, 61). However, this therapy only benefits a small number of patients (62). Therefore, it is important to develop new strategies to reshape the immunosuppressive TME. As a proinflammatory form of cell death, it is promising to induce pyroptosis of tumor cells in local tumor tissue. In this study, after the tumor was fully established, we directly administered the pyroptosis-inducible cells into tissue to test the potency of this strategy to locally elicit antitumor immunity and modify the tumor immune microenvironment. Our results supported previous findings that the induction of tumor ICD in just a few tumor cells was sufficient to elicit antitumor immune responses and suppress the tumor growth (52, 53, 63). The responses of antitumor effector cells, including adaptive CTLs and innate NK cells, were increased in the spleen and tumor tissue, while the immunosuppressive MDSCs were significantly reduced. These results indicated that local tumor delivery and induction of GSDMD-NT expression and pyroptosis of pyroptosis-inducible cells presented a promising intratumor immunotherapy strategy. Although intratumoral delivery of chemical drugs for ICD induction might be a clinical option for the treatment of some cancers (64), our strategy has some advantages. Chemotherapeutics generally require a very high dose to induce significant ICD, which raises severe safety concerns considering the side effects of chemotherapeutics. In contrast, our approach only needs the use of tetracycline or its analogs, which are clinically applicable, to induce GSDMD-NT expression.

Another possible optional strategy for clinically inducing tumor pyroptosis is to directly target GSDM family proteins and stimulate their expression and processing specifically in tumor cells. However, if pyroptosis is induced in normal cells expressing GSDMs, severe side effects might be caused due to damage to the normal functions of these cells, as well as the generation and release of a large amount of inflammatory mediators. In fact, GSDME is expressed in many normal tissues but is silenced in most cancer cells. After caspase-3 is activated by chemotherapeutics or GSDME is targeted for regulation, normal cells may show GSDME-dependent pyroptosis (26, 54). In contrast, our strategy does not aim to activate endogenous GSDMs and just induces tumor cell pyroptosis in a strictly controlled way just in the genetically modified cells. In addition, the number of pyroptotic tumor cells is also a key factor that should be controlled. Excessive pyroptosis and the release of inflammatory factors may lead to excessive or abnormal activation of the immune system, leading to secondary pyroptosis of normal cells, and ultimately to systemic inflammation (65, 66). It is difficult to control the number of tumor cells undergoing pyroptosis using chemotherapeutic drugs or other pyrolysis inducers, while the approach reported here can control the intensity of pyroptosis by adjusting the number of modified GSDMD-NT tumor cells.

Returning to classical vaccine applications, we further used pyroptosis-inducible tumor cells to immunize tumor-bearing mice, which simulates a clinical setting of vaccine treatment. Immunization led to significant inhibition of the growth of the established contralaterally inoculated tumor. Similar to local delivery in tumors, distal subcutaneous immunization of modified cells also significantly stimulated enhanced antitumor CTL and NK responses and suppressed immunosuppressive MDSC and Treg responses in both spleen and tumor tissues. In this study, mice were given 5% sucrose water with doxy at a high dose of 500 μg/mL for 2 days to guarantee reaching at an effective working concentration quickly and then at a dose of 20 μg/mL (i.e. 8-12µg/g body weight per day) to maintain the induction of GSDMD-NT expression constantly in the following days. Although we didn’t carefully test the most appropriate or minimum doses of doxy for inducing GSDMD-NT expression *in vivo*, our data showed that the dose 8-12 µg/g body weight per day worked well in mice, which preliminarily implied the applicability of the induction practice for human referring to the recommended dose of oral tetracycline for adults is about 0.75g to 1.5g/day, and that of intravenous infusion is 1 to 1.5g/day (roughly 14 to 21 µg/g body weight).

Taken together, we developed a new tumor cell vaccine strategy that adequately takes advantage of the immunological characteristics and broad-spectrum tumor antigens of ICD tumor cells. We confirmed that overexpression of the key pyroptosis effector molecule GSDMD-NT was capable of directly inducing pyroptosis and presenting robust immune stimulation. Employing preventive or therapeutic immunization strategies including direct delivery to local tumors, we revealed that a pyroptosis-inducible cell vaccine produced robust antitumor immunity and effects. Considering that there is a huge demand for personalized tumor treatment (67) due to the instability of the tumor cell genome, the strategy described here represents a new and promising personalized clinical vaccine strategy. Notably, this strategy can be further explored by transgenic modification with clinically identified tumor neoantigens or extra immunoregulating genes to elicit more forceful and effective immune responses.

## Experimental model and subject details

### Mice and cell lines

Male C57BL/6 mice (6-8 weeks, 16~18 g) and female or male BALB/c mice (6-8 weeks, 16~18 g) were provided by the Central Animal Care Services of the Institute of Medical Biology, Chinese Academy of Medical Sciences (CAMS) and Peking Union Medical College (PUMC, Kunming, China). All mice were maintained in a specific pathogen-free environment. The animal procedures were performed with ethical compliance and approval by the Animal Care and Welfare Ethics Committee, Institute of Medical Biology, CAMS. TC-1 cells are lung epithelial cells of C57BL/6 mice cotransfected with HPV-16 E6 and E7 and the c-Ha-ras oncogene. 4T1 cells are breast cancer cells of BALB/c mice purchased from the tumor Center of the Chinese Academy of Medical Sciences. 293T/CT26 cells were purchased from the American Type Culture Collection (ATCC). TC-1/4T1/CT26 cells were cultured in Roswell Park Memorial Institute (RPMI) 1640 media (Servicebio), and 293T cells were cultured in Dulbecco’s modified Eagle’s medium (Servicebio). Cells were cultured in media containing 10% fetal bovine serum (FBS, Gibco) and 1% penicillin–streptomycin (BI) at 37 °C in 5% CO2.

### Strains and plasmids

The mouse-derived GSDMD-NT gene (amino acids 1-276) and eGFP gene were separately cloned into the pLVX-TetOne-Puro vector (MiaoLing Plasmid Platform) to obtain recombinant plasmids. The pLVX-TetOne-Puro vector, pLVX-TetOne-Puro-GSDMD-NT, and pLVX-TetOne-Puro-eGFP constructs and lentivirus packaging plasmid (psPAX2, pMD2. G) were transformed into E. coli Stbl3 (MiaoLing Plasmid Platform). Strains were cultured in lysogeny broth (LB) medium with the appropriate antibiotics (ampicillin: 100 μg/mL) in a 37 °C shaking incubator.

## Method details

### Lentivirus production

The appropriate lentivirus packaging kit was purchased from Invitrogen (Lipofectamine™ LTX Reagent with PLUS™ Reagent, REF: 15338100) and used according to the manufacturer’s guidelines. Harvested viral particles (lenti-Vector, lenti-GSDMD-NT, lenti-eGFP) were added to TC-1, 4T1 or CT26 cells. After 24 hours, the cells were washed with RPMI 1640 media and puromycin (5 μg/mL) was added to screen for stably expressed cell lines. The selected cell lines were maintained with puromycin at a concentration of 2.5 μg/mL

### RNA extraction and real-time qPCR

Total RNA was extracted from the cells and tissues using TRIzol reagent (Invitrogen, Carlsbad, China) followed by DNase I digestion, according to the manufacturer’s instructions. RNA was quantified by measuring the absorbance at 260 nm and reverse transcription into cDNA (PrimeScript™ RT Reagent Kit gDNA Eraser, Takara). Real-time quantitative PCR (qPCR) was performed using the Bio-Rad IQ5 real-time PCR system according to the manufacturer’s instructions (ChamQ Universal SYBR qPCR Master Mix, Vazyme). The primers used for qPCR are shown in **Supplementary Table 1**. The data were normalized using endogenous β-actin mRNA. The 2−ΔΔCt method was used to analyze the PCR data.

### Western blotting

Samples were lysed in RIPA buffer (Solarbio) containing 1% (v/v) protease inhibitor. The protein concentration for each sample was detected using a BCA protein concentration determination kit (Beyotime Biotechnology) in accordance with the manufacturer’s recommendations. Subsequently, the whole-cell lysates or cell supernatant were separated by SDS–PAGE and transferred to PVDF membranes (Millipore). Rabbit monoclonal anti-GSDMD antibody (ab209845; 1:1000), rabbit monoclonal anti-HMGB1 antibody (ab79823; 1:10000) and mouse monoclonal β-actin antibody (ab6276; 1:5000) from Epitomics (Abcam, Cambridge, MA) were incubated in 1×TBST containing 5% milk for 2 h at room temperature (RT) with continuous shaking. After washing with 1×TBST three times for 10 min/wash at RT, the membranes were incubated with horseradish peroxidase (HRP)-conjugated goat anti-rabbit secondary antibody (ab6721; 1:10000) or HRP-conjugated goat anti-mouse secondary antibody (ab6789; 1:5000) from Epitomics (Abcam, Cambridge, MA) for 2 h at RT. After a final washing step with 1×TBST three times for 10 min/wash at RT, the immunoreactive bands were visualized by enhanced chemiluminescence (ECL) (Thermo Fisher Scientific, San Jose, CA) for the indicated exposure times.

### Immunofluorescence

TC-1-GSDMD-NT cells on the slides were collected and treated with 4% paraformaldehyde containing 0.1% Triton X-100 for 10 min, followed by three washes with PBS. Then, the cells were blocked with 2% BSA in PBS for 1 h and incubated with rabbit polyclonal anti-GSDMD antibody (Affinity, AF4012; 1:100 in 2% BSA) for 2 h at RT, followed by three washes with PBS. Next, the samples were incubated with FITC-conjugated secondary antibody (1:2,000 in 1% BSA, Proteintech) for 2 h at RT. After washing five times with PBS, the nuclei were stained with DAPI (ab104139, Abcam) for 10 min. All of the samples were examined by confocal microscopy. Tumor tissues were collected and fixed in formalin, embedded in paraffin, and sectioned. After deparaffinization and hydration, the slides were immersed in EDTA antigen retrieval buffer. Then, the slides were incubated with anti-CD4 antibody (Biolegend) and anti-Foxp3 antibody (Biolegend) or rabbit polyclonal anti-GSDMD antibody (Affinity, AF4012, 1:100) overnight at 4 °C. Next, Cy3-conjugated secondary antibody (Servicebio, GB21303; 1:2,000 in 2% BSA) was added, followed by incubation at room temperature for 1 h. Then, the slides were incubated with DAPI (ab104139, Abcam) at RT for 10 min. All of the sections were examined by fluorescence microscopy.

### Cell proliferation assay, apoptosis and the toxicity of doxy assays

For the proliferation curve, the cells were incubated in a 96-well plate (2×10_3_ TC-1 cells or CT26 cells, 1×10_3_ 4T1 cells). At 0 h, 24 h, 48 h, 72 h and 96 h after seeding, 10% CCK-8 (Solarbio) was added and incubated for 2 h, followed by absorbance measurement at 450 nm.

For apoptosis analysis, cells were seeded into 6-well plates (5×10_5_ cells per well). After 24 h, 10 mg/mL doxy was added to the media until the final concentration was 1 μg/mL. Then, the sample was collected and stained using an apoptosis test kit following the manufacturer’s recommendations (Becton, Dickinson and Company). Then, the samples were analyzed by flow cytometry.

For the toxicity of doxy assays, we prepared 96-well plates as for the cell; proliferation assay and added CCK-8 to measure the absorbance. The only difference was due to the expression of GSDMD-NT causing pyroptotic cell death.

The release of LDH, ATP and inflammatory cytokines after inducing pyroptosisA total of 5×10_4_ cells were incubated in a 12-well plate for 24 h and doxy was added to induce cell death. At the indicated time points, the supernatant was collected and cleared from the dead cells by centrifugation.

For LDH release assays, the positive stimulator was added one hour before medium collection, and LDH was quantified with the LDH Cytotoxicity Assay Kit in accordance with the manufacturer’s recommendations (Beyotime).

For the ATP release assays, the supernatant was either used immediately or frozen at -20 °C for later use. ATP was measured using the Enhanced ATP Assay Kit in accordance with the manufacturer’s recommendations (Beyotime).

To detect inflammatory cytokines, the supernatant was collected after inducing for 8 h with doxy and quantified with the IL-6 ELISA kit (Invitrogen), IL-1β ELISA kit (Invitrogen) and IL-18 ELISA kit (Invitrogen).

### Generation, maturation and migration of mouse BMDCs

BMDCs were extracted from the femurs and tibias of 7-week-old C57BL/6 mice and cultured for 8 days. Briefly, bone marrow cells were incubated in RPMI-1640 medium with 10% FBS and recombinant granulocyte-macrophage colony-stimulating factor (GM-CSF, 20 ng/mL, PeproTech). On the 5th day, the medium was replaced with fresh medium with GM-CSF, and after 2 days the BMDCs were harvested for future use.

For the BMDC maturation assay, TC-1 cells (vector, NT-GSDMD) and BMDCs were cocultured in a 6-well plate at a ratio of 1:10 for 24 h, and doxy was added to induce pyroptotic cell death. The positive control BMDCs were cocultured with LPS (100 ng/mL). Next, the cocultured cells were harvested and stained with CD11c, CD86, CD80, MHC-I and MHC-II (Biolegend). Finally, flow cytometry was used to analyze the percentage of mature BMDCs.

For the BMDC migration assay using Transwells, GSDMD-NT-TC-1 cells were untreated or induced to undergo pyroptotic cell death with doxy for 24 h. Then, the supernatant was collected and placed in the bottom chambers of a 24-well Transwell plate and BMDCs were added to the upper chamber. The migration rate of the BMDCs was measured by optical microscopy after incubation for 6 h.

### Mouse xenograft tumor models

To induce pyroptotic tumor models *in vivo*, C57BL/6 or BALB/c mice were subcutaneously injected with 1×105 GSDMD-NT-TC-1 cells or GSDMD-NT-CT26 cells. When the tumor grew to a certain size, pyroptosis of the tumor cells was induced by putting doxy in the animals’ drinking water.For the tumor treatment model, C57BL/6 mice were subcutaneously injected with 1×105 TC-1 cells into the right flank and when the tumor volume reached approximately 60 mm3, the mice were treated with subcutaneous injections of 1×106 GSDMD-NT/Vector-TC-1 cells, FT-cell lysates or PBS on the left flank or the local site of the primary tumor, once every three days. Pyroptosis of the tumor cells was induced by supplying doxy in the drinking water on the day of inoculation.

In the preventive tumor models, C57BL/6 or BALB/c mice were subcutaneously injected with 1×105/1×106/1×107 GSDMD-NT-TC-1 cells, FT-cell lysates, or PBS, and pyroptosis of tumor cells was induced by putting doxy in their drinking water on the day of inoculation. Then, 1×105 TC-1 or CT26 cells were subcutaneously inoculated after one week, followed by tumor growth measurements and the time of tumor appearance records.

Tumor volumes were measured with a caliper (length (a), width (b), and height (h)) and calculated as follows: tumor volume = a×b×h/2. The tumors were excised and weighed, and immune cells in the spleens and tumors were examined by flow cytometry. The tumor tissue was fixed and subjected to immunofluorescence and TUNEL assays.

### Methods of inducing pyroptosis *in vitro* and *in vivo*

The half-life of doxy in cell culture medium is 24 h. To maintain continuous induction of the GSDMD-NT/eGFP gene in cell culture, the medium was replenished with doxy every 48 h.

*In vitro*: The final concentration of doxy was 1 μg/mL in cell culture.

*In vivo*: Mice were given 5% (m/v) sucrose water with doxy at a high dose (500 μg/mL) for 2 days to reach its effective concentration quickly and then given at a low dose (20 μg/mL) to maintain its effect constantly.This regimen of doxy administration can achieve a quick switch between ON and OFF statuses of tTA-activated GSDMD-NT gene expression to induce pyroptosis.

### Flow cytometry

Approximately 0.5 g of tumor tissue was digested in collagenase A (Sigma) at 37 °C at 170 rpm for 30 min. Lymphocytes were isolated from spleens or the digested tumor tissue by mashing it through a 70 μM strainer and using lymphocyte isolation solution (Biolegend). Then, the lymphocytes were plated into 96-well U-plates at 1×106 cells per well. The cells were collected for staining according to the manufacturer’s protocols (BioLegend, Inc., San Diego, CA, USA). They were stained with APC-anti-mouse CD8α, PE-anti-mouse IFN-γ, APC-anti-mouse CD11b, PE-anti-mouse Gr-1, PE-anti-mouse Foxp3, APC-anti-mouse CD4, FITC-anti-mouse CD25, PE-anti-mouse NK1.1, FITC-anti-mouse CD45, and the HPV16 CTL peptide epitope E749-57 (49 RAHYNIVTF57), which was synthesized at >98% purity by GL Biochem Ltd. (Shanghai, China).

CTLs need the E749-57 peptide (5 μg/mL) to stimulate tumor antigen-specific CD8+ T cells to produce IFN-γ. Finally, the data were acquired with BD AccuriC6 (BD Biosciences) and analyzed with FlowJo.

### Enzyme-linked immunospot (ELISPOT) assays

A total of 3×105 lymphocytes from tumors or spleens were seeded into a 96-well ELISPOT plate, and the quantification of the IFN-γ spots was assessed with an IFN-γ ELISPOT assay kit (MABTECH). The E749-57 peptide (5 μg/mL) was used to stimulate tumor antigen-specific CD8+ T cells to produce IFN-γ for 24 h. The spots were photographed with an ELISPOT Reader System (AID Diagnostika GmbH, Strasberg, Germany).

### TUNEL assays

The sections were derived from the paraffin-embedded tumor tissues, dewaxed and repaired with citric acid antigen repair buffer. Then, 3% (m/v) bovine serum albumin (BSA) was used to block the samples, followed by incubation with the primary antibodies at 4 °C overnight. Next, the secondary antibodies were added and incubated at room temperature (RT) for 50 min in a dark environment. The samples were treated with the TUNEL reaction solution according to the manufacturer’s protocol (Servicebio, G1501) and DAPI. Finally, a fluorescence microscope was used to view the samples.

## Quantification and statistical analysis

Statistical analysis was performed with GraphPad Prism 8.0 software and Excel 2020. All of the values in the present study are presented as the means ± SD. The differences in tumor growth were analyzed by two-way ANOVA followed by Tukey’s multiple comparison test. Unpaired Student’s t-test was used for two-group comparisons, and one-way ANOVA was used for other multiple comparisons when more than two groups were compared. Significant p values are denoted by *p value < 0.05, **p value < 0.01, ***p value < 0.001, ns represents no significance.

## Data availability statement

The original contributions presented in the study are included in the article/**Supplementary Material**. Further inquiries can be directed to the corresponding author.

## Ethics statement

This study was reviewed and approved by the Animal Care and Welfare Ethics Committee, Institute of Medical Biology, CAMS (ethics number: DWSP201805008).

## Author contributions

JH, PZ and YC were also responsible for designing and performing the experiments, analyzing the data, and writing the paper. JQ, CY, DL, YY (7^th^ author), YY (8^th^ author), QWL, YH, XZ, WL, LH, ZY, HC, WS, XY, QL, HB and WH participated in performing animal experiments. YM conceived and supervised the project. All authors contributed to the article and approved the submitted version.

## Acknowledgments

We are grateful for the financial support of the Chinese Academy of Medical Sciences (CAMS) Innovation Fund for Medical Sciences (CIFMS) (grant number 2021-I2M-1-043), the Science and Technology Project of Yunnan Province (202002AA100009), and the National Natural Science Foundation of China (82073371).

## Conflict of interest

The authors declare that the research was conducted in the absence of any commercial or financial relationships that could be construed as a potential conflict of interest.

## Publisher’s note

All claims expressed in this article are solely those of the authors and do not necessarily represent those of their affiliated organizations, or those of the publisher, the editors and the reviewers. Any product that may be evaluated in this article, or claim that may be made by its manufacturer, is not guaranteed or endorsed by the publisher.

## Supplementary material

Supplementary Figure 1 Western blotting analysis on the expression of endogenous full length GSDMD and exogenous GSDMD-NT in **(A)** 4T1 and **(B)** CT26 cells.

Supplementary Figure 2 Inducible GSDMD-NT overexpression induces the significant suppression on the orthotopically transplanted and fully established 4T1 breast tumor with genetically modified tumor cells in BALB/c mice. **(A)** The protocol; **(B)** the size of isolated tumor masses; **(C)**The monitoring of tumor growth;**(D)** tumor weight; **(E)** spleen weight; **(F)** flow cytometry analysis on CD8^+^IFN-γ^+^ splenocytes; **(G)** flow cytometry analysis on CD11b^+^Gr-1^+^ splenocytes.

Supplementary Figure 3 Tumor growth curves by each mouse in TC-1 tumor models (n = 12).

Supplementary Figure 4 Tumor growth curves by each mouse in CT26 tumor models (n = 10).

Supplementary Figure 5 Tumor growth curves by each mouse in TC-1 tumor models (n = 6).

Supplementary Figure 6 Tumor growth curves by each mouse in TC-1 tumor models (n = 8).
