# Supplementary figures and images for: A new personalized vaccine strategy based on inducing the pyroptosis of tumor cells in vivo by transgenic expression of a truncated GSDMD N-terminus
Source: Front Immunol. 2022 Sep 15;13:991857. doi: 10.3389/fimmu.2022.991857 (PMC9521720; doi:10.3389/fimmu.2022.991857)

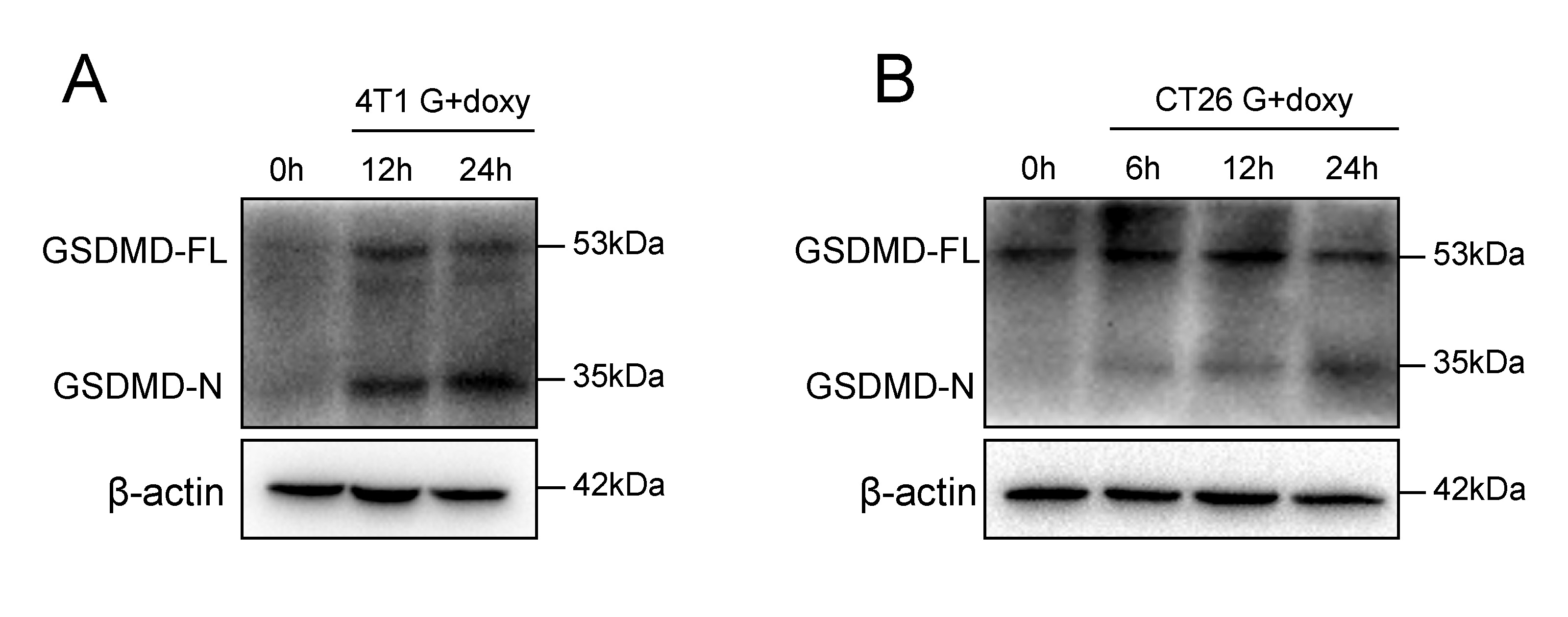

Supplement: Supplementary Figure 1 — Western blotting analysis on the expression of endogenous full length GSDMD and exogenous GSDMD-NT in (A) 4T1 and (B) CT26 cells. [file Image_1.jpeg]

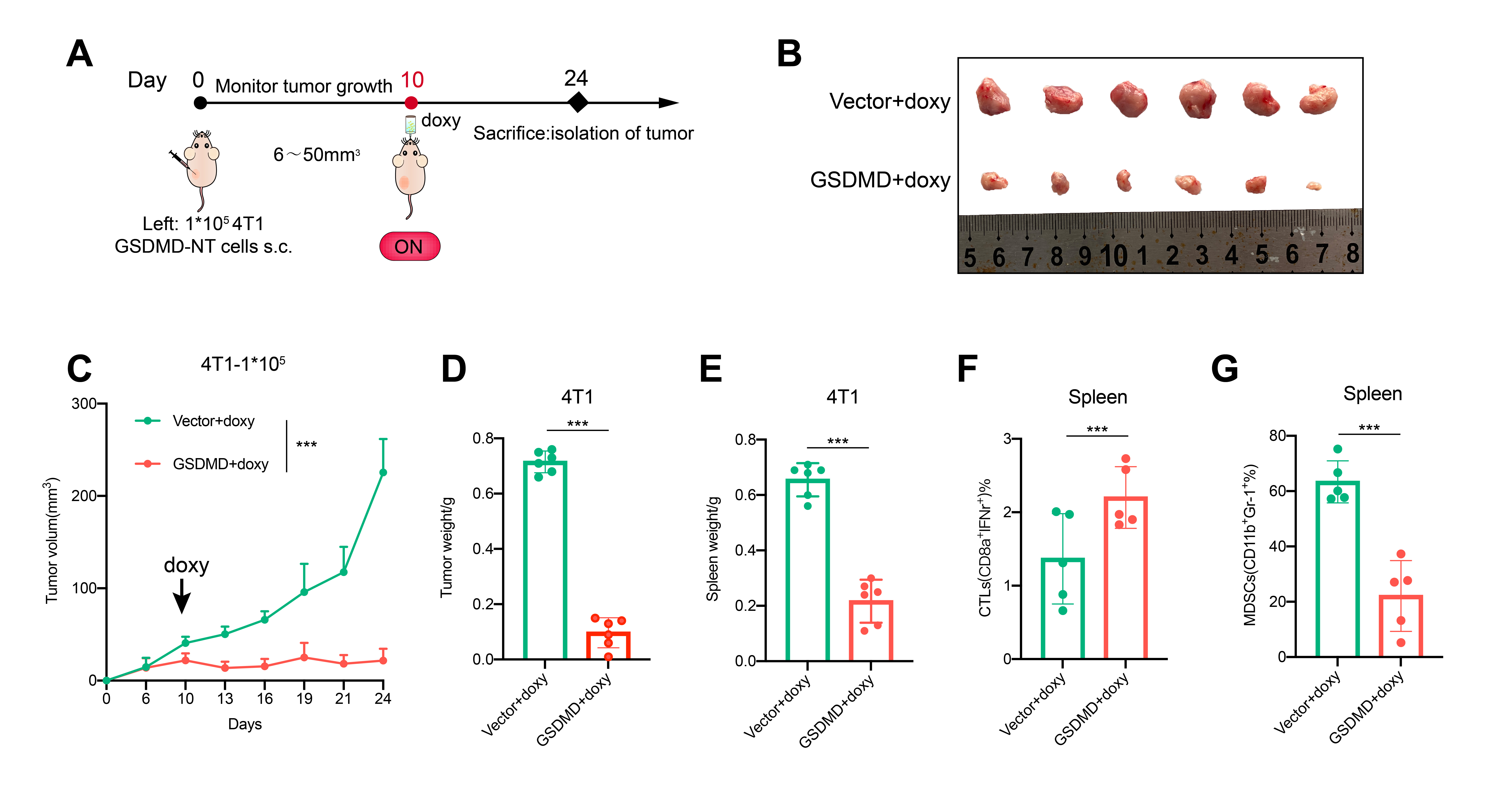

Supplement: Supplementary Figure 2 — Inducible GSDMD-NT overexpression induces the significant suppression on the orthotopically transplanted and fully established 4T1 breast tumor with genetically modified tumor cells in BALB/c mice. (A) The protocol; (B) the size of isolated tumor masses; (C)The monitoring of tumor growth;(D) tumor weight; (E) spleen weight; (F) flow cytometry analysis on CD8+IFN-γ+ splenocytes; (G) flow cytometry analysis on CD11b+Gr-1+ splenocytes. [file Image_2.jpeg]

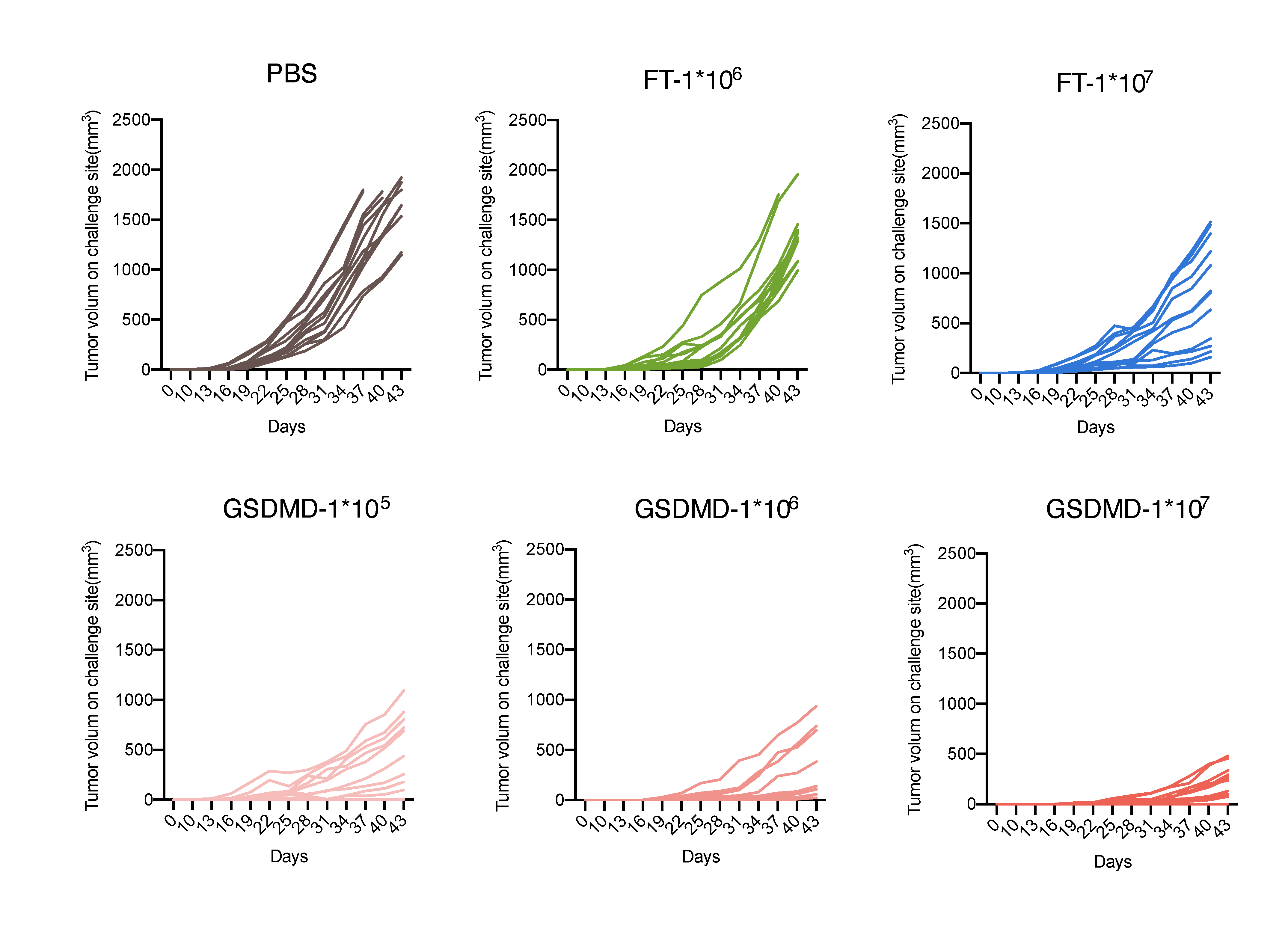

Supplement: Supplementary Figure 3 — Tumor growth curves by each mouse in TC-1 tumor models (n = 12). [file Image_3.tif]

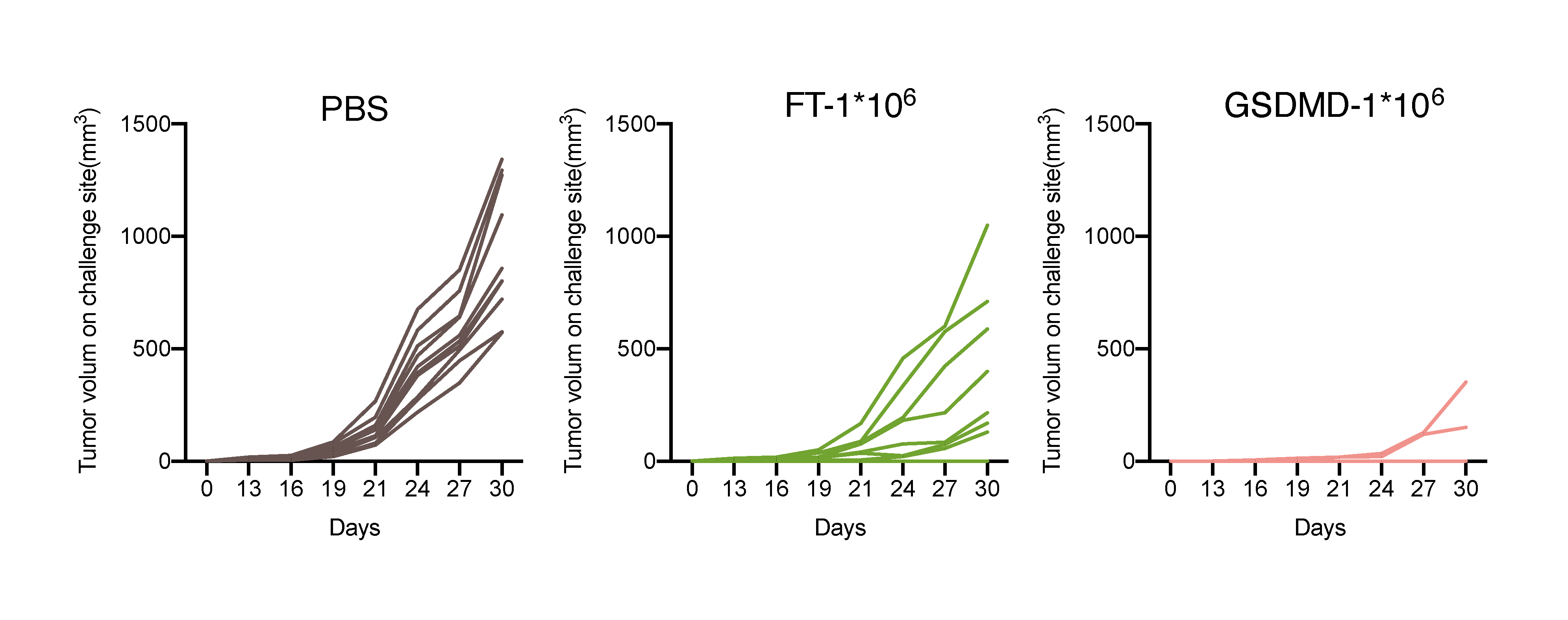

Supplement: Supplementary Figure 4 — Tumor growth curves by each mouse in CT26 tumor models (n = 10). [file Image_4.tif]

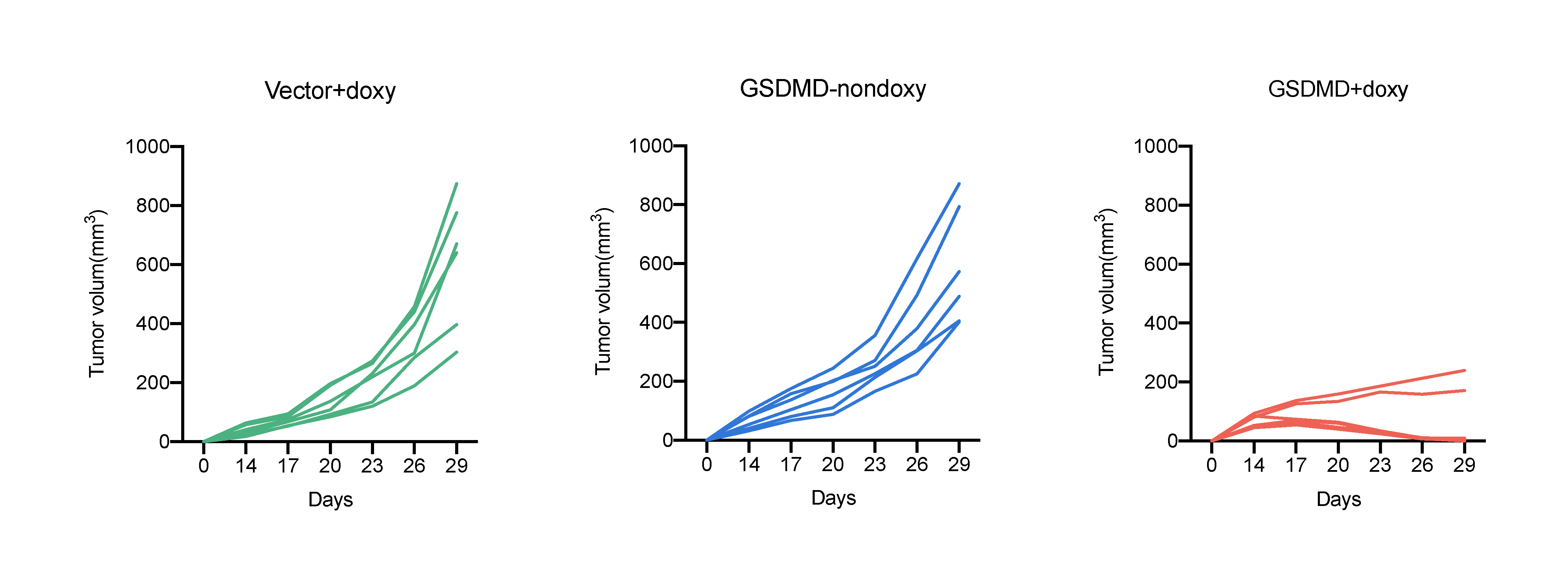

Supplement: Supplementary Figure 5 — Tumor growth curves by each mouse in TC-1 tumor models (n = 6). [file Image_5.tif]

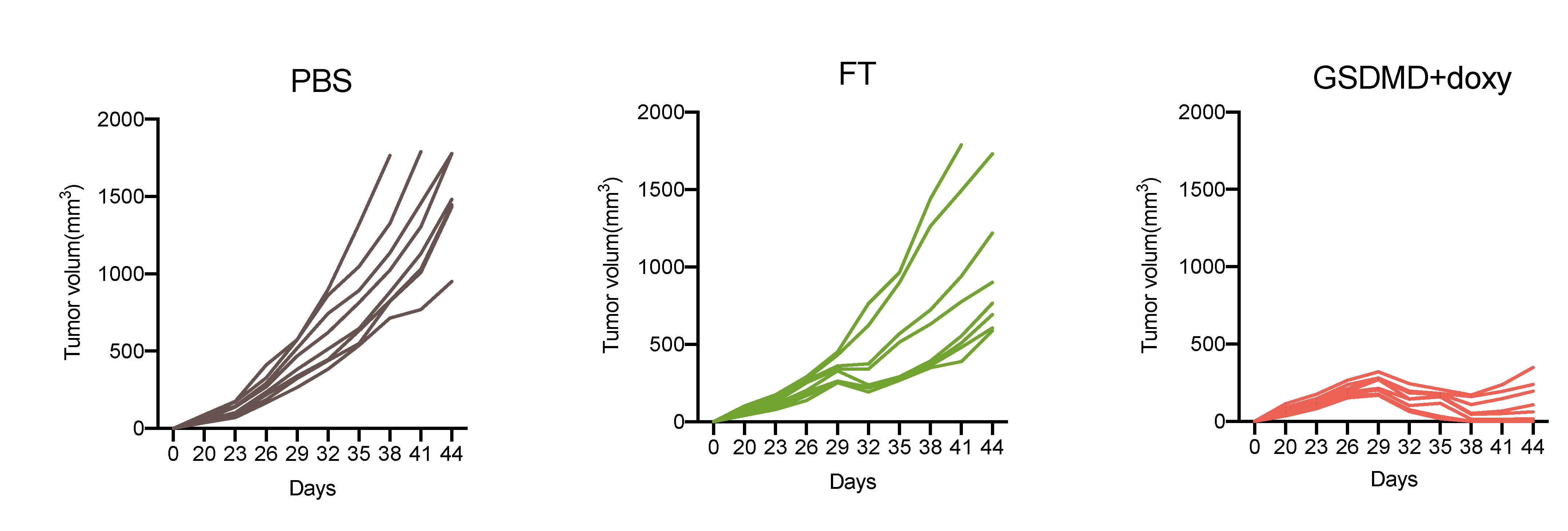

Supplement: Supplementary Figure 6 — Tumor growth curves by each mouse in TC-1 tumor models (n = 8). [file Image_6.tif]
