# Supplementary material for: A new personalized vaccine strategy based on inducing the pyroptosis of tumor cells in vivo by transgenic expression of a truncated GSDMD N-terminus
Source: Front Immunol. 2022 Sep 15;13:991857. doi: 10.3389/fimmu.2022.991857 (PMC9521720; doi:10.3389/fimmu.2022.991857)
Supplement: Supplementary file 7 [file Table_1.docx]

Supplementary table 1: primers sequence used in this paper

| Primers | Sequence |
| --- | --- |
| GSDMD-NT-F | CAGCAGGCAGTAGGGTCT |
| GSDMD-NT -R | GAGAAGGTGGTCAAGAACG |
| HSP70-F | CGACCTGGGCACCACCTACT |
| HSP70-R | CCACCGCATCGCCGAACTT |
| HSP90-F | TAAGTTGGACAGTGGGAAA |
| HSP90-R | CGAGTAGAATCCGACACC |
| H-2K^b^-F | GAGTATTGGGAGCGGGAGA |
| H-2K^b^-R  β-actin-F  β-actin-R | TCGTTCAGGGCGATGTAA  ACACCCGCCACCAGTTCGC  ATGGGGTACTTCAGGGTCAGGATA |
